# Comparative Vector Analysis of Astigmatic Changes After Small-incision Lenticule Extraction versus Femtosecond Laser In -situ Keratomileusis

**DOI:** 10.18502/jovr.v21.18010

**Published:** 2026-07-15

**Authors:** Mai Hong Phan, Luong Minh Huynh, Say Kiang Foo

**Affiliations:** ^1^Ho Chi Minh City Eye Hospital, Ho Chi Minh, Vietnam; ^2^SEGi University, Petaling Jaya, Malaysia

**Keywords:** Astigmatism, LASIK, Myopia, SMILE, Vector Analysis

## Abstract

**Purpose:**

To compare thevisual outcomes of small-incision lenticule extraction (SMILE) versus femtosecond laser in-situ keratomileusis (FS-LASIK) in eyes with myopia and high astigmatism.

**Methods:**

This retrospective study was conducted at a tertiary eye hospital between January 2021 and December 2022. Data were collected from individuals with myopia up to - 10.00 diopters (D) and cylinder ≥ 2.50 D who underwent SMILE (VisuMax 500) or FS-LASIK (MEL 90) and completed a 3-month postoperative follow-up.

**Results:**

A total of 170 eyes of 123 patients were included: 83 eyes (60 patients) underwent SMILE, and 87 eyes (63 patients) underwent FS-LASIK. The preoperative cylindrical refractive error was - 3.01 ± 0.61 D in the SMILE group and - 3.09 ± 0.66 D in the FS-LASIK group. At 3 months, there was no statistically significant difference in uncorrected visual acuity (UCVA) or manifest refraction spherical equivalent (SEQ) between the two groups (*P *= 0.26 and *P *= 0.88, respectively). In the SMILE group, 90.4% of eyes achieved an uncorrected distance visual acuity (UDVA) of 20/20 or better compared to 98.77% with a preoperative corrected distance visual acuity (CDVA) of 20/20 or better. In the FS-LASIK group, 80.46% of eyes achieved a UDVA of 20/20 or better compared to 94.25% with a preoperative CDVA of 20/20 or better. Moreover, 95.1% of eyes in the SMILE group and 94.3% in the FS-LASIK group were within ± 0.50 D. Vector analysis showed comparable results between the two groups in terms of target-induced astigmatism (*P * = 0.559), difference vector (*P * = 0.155), index of success (*P * = 0.091), and angle of error (*P * = 0.89). The surgically induced astigmatism and correction index were significantly lower in the SMILE group (*P * < 0.01), whereas the absolute magnitude of error was higher in the SMILE group (*P * = 0.02).

**Conclusion:**

SMILE and FS-LASIK have comparable efficacy, predictability, and safety in correcting myopia associated with high astigmatism ( ≥ 2.50 D).

##  INTRODUCTION

It has been more than 30 years since laser in-situ keratomileusis (LASIK) was first introduced.^[1]^ In LASIK surgery, a corneal flap is created, and then an excimer laser ablation is performed on the underlying corneal stroma to reshape the tissue. LASIK is a widely adopted surgical procedure for the correction of refractive errors, with excellent safety and efficacy.^[2,3,4,5,6]^ It is estimated that around 25 million eyes have been treated in the US over the last 25 years.^[7]^ More recently, small-incision lenticule extraction (SMILE) received CE mark approval. Performed using the VisuMax 500 femtosecond laser (Carl Zeiss Meditec AG, Jena, Germany), SMILE enables vision correction without the need for a corneal flap. It reshapes the cornea by separating a piece of tissue lenticule, which is then removed using a small laser incision. The first clinical results of this procedure were reported by Sekundo et al and Shah et al.^[8,9,10]^ Since then, SMILE has become one of the most preferred choices in the treatment of myopia and myopic astigmatism,^[11,12]^ with more than 9 million procedures performed to date (Personal communication with Carl Zeiss Meditec, May 10, 2024).

Studies comparing the visual outcomes of astigmatism correction with SMILE and LASIK (mostly < 2.50 D of cylinder) have shown slightly better outcomes with LASIK.^[9,13,14]^ However, few studies have conducted in-depth postoperative astigmatism analyses. The precision of astigmatism correction in SMILE is mixed within the literature.^[15,16,17]^ It is necessary to accurately evaluate the efficacy of additional analysis of astigmatism treatment beyond the standard nine graphs.^[18]^ The most commonly used analysis is the Alpins method,^[19]^ which incorporates vectors such as surgically induced astigmatism (SIA), target-induced astigmatism (TIA), and difference vector (DV) to examine the efficacy of astigmatism correction.

This study aimed to compare theoverall visual outcomes and perform a comprehensive astigmatism analysis to evaluate astigmatism correction after SMILE and femtosecond laser in-situ keratomileusis (FS-LASIK) in patients with myopia and high astigmatism.

##  METHODS

This retrospective study included all patients who underwent SMILE or FS-LASIK for myopic astigmatism between January 2021 and December 2022 at the Excimer Center of Ho Chi Minh City Eye Hospital (Ho Chi Minh City, Vietnam) and who completed a 3-month postoperative follow-up.

The inclusion criteria were: eligibility for SMILE or FS-LASIK, age ≥ 20 years, myopia up to - 10.00 D, astigmatism of at least 2.50 D, stable refraction over the past 12 months (a change within ± 0.50 D was acceptable), healthy cornea, and an estimated residual stromal thickness of at least 280 µm for FS-LASIK or a combined residual stromal thickness and cap thickness of ≥ 400 µm for SMILE.

Patients with other significant ocular pathologies, neurological or systemic diseases, preoperative corrected distance visual acuity (CDVA) worse than 0.1 logMAR (logarithm of the Minimum Angle of Resolution; equivalent to Snellen 20/25 or poorer), dry eye syndrome, corneal epitheliopathy, or a history of inflammatory corneal disease were excluded.

Following detailed counseling on the procedural characteristics, potential outcomes, and associated risks of each technique, all patients made an informed choice between SMILE and FS-LASIK and signed an informed consent form before surgery. Ethics approval for this retrospective data analysis was granted, and the study was conducted in compliance with the tenets of the Declaration of Helsinki.

### Preoperative Assessment

The preoperative assessment included uncorrected distance visual acuity (UDVA), CDVA, intraocular pressure (IOP) by air-puff tonometer (CT-800, Topcon, Tokyo, Japan), slit-lamp biomicroscopy, subjective manifest and cycloplegic refraction, as well as corneal topography and central corneal thickness using Pentacam (Oculus Optikgeräte GmbH, Wetzlar, Germany).

#### Surgical Protocol

All operations were performed by an experienced surgeon (MHP) to ensure consistency of the surgical technique. All eyes were targeted for emmetropia. In the FS-LASIK group, flaps were created using the VisuMax 500 femtosecond laser (Carl Zeiss Meditec AG, Jena, Germany). All flaps had a superior hinge, a thickness of 90 to 100 μ m, and a diameter of 8.1 to 8.8 mm. An excimer laser (MEL90, Carl Zeiss Meditec AG, Jena, Germany) was used for tissue ablation. Optical zones ranged from 6.0 to 6.5 mm. In the SMILE group, all treatments were performed using the VisuMax 500 femtosecond laser. Parameters for the femtosecond laser included a lenticule diameter of 6.0 to 6.5 mm, a cap diameter of 7.0 to 7.5 mm, a cap thickness of 100 μ m, and a single 2-mm small incision at 125º.

In the FS-LASIK group, all eyes with a cylinder were marked at the limbus at 0º and 180º while the patient was in an upright position. Subsequently, once the patients assumed a supine position, the surgeon secured the position of the eye with two toothed forceps, aligning with the two limbal marks superimposed with a zero line on the Mel 90 microscope to avoid cyclotorsion or patient noncompliance. The FS-LASIK treatment nomogram was adjusted to include a 0.50 D spherical overcorrection and full astigmatic correction.

In the SMILE group, all eyes with cylinders were similarly marked at the limbus at 0º and 180º while the patient was upright. In the supine position, the cornea was marked again from the limbal marks toward the center of the pupil using a Rumex thin hockey hook to further ensure visibility within the cone. After docking, the cone was manually rotated to align the corneal marks with the horizontal axis displayed on the observational screen of the VisuMax 500. The SMILE nomogram incorporated an overcorrection of 0.75 diopters (D) for spherical refractive errors and provided full correction for cylindrical errors.

### Postoperative Evaluation

As part of routine care, patients were seen on day 1, week 1, month 1, and month 3 postoperatively. They were instructed to follow the standard care and medication regimen, which included Cravit 0.5% (Santen) and Pred Forte 1% (Allergan) four times daily for 1 week after treatment. Manifest refraction and visual acuity measurements (monocular UDVA and CDVA), as well as corneal topography, were performed at the 3-month postoperative visit.

**Table 1:** Patient demographics

	**SMILE**	**FS-LASIK**	***P*** **-value**
Eyes (patients)	83 (60)	87 (63)	
Sex (Male/Female), *N*	15/45	13/50	
Age (years), mean ± SD (range)	24.9 ± 5.2 (18 to 41)	23.5 ± 3.60 (18 to 35)	0.24
SEQ (D), mean ± SD	- 4.91 ± 1.81	- 5.87 ± 2.25	< 0.01
Sphere (D), mean ± SD (range)	- 3.44 ± 1.91 (0 to - 7.00)	- 4.33 ± 2.40 (0 to - 7.50)	< 0.05
Cylinder (D), mean ± SD (range)	- 3.01 ± 0.61 ( - 2.50 to - 4.75)	- 3.09 ± 0.66 ( - 2.50 to - 4.75)	0.559
UDVA (logMAR), mean ± SD	1.06 ± 0.25	1.13 ± 0.25	0.062
CDVA (logMAR), mean ± SD	0.01 ± 0.02	0.01 ± 0.03	0.12
Keratometry (delta K) (D), mean ± SD	3.09 ± 0.77	3.13 ± 0.68	0.731
Central corneal thickness (microns), mean ± SD	548.64 ± 32.11	535.61 ± 23.93	< 0.01
D, diopters; SD, standard deviation; SEQ, spherical equivalent; UDVA, uncorrected distance visual acuity; CDVA, corrected distance visual acuity.			

**Figure 1:**
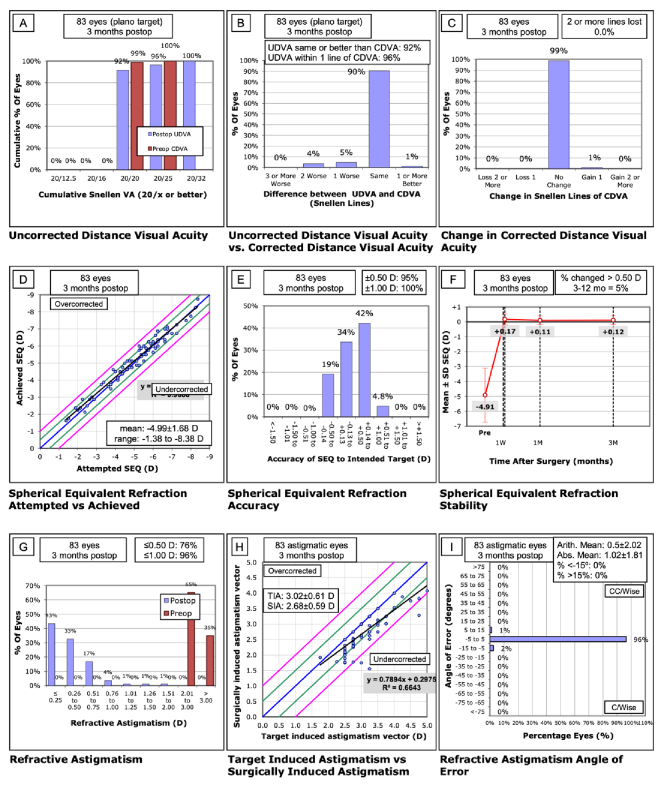
Standard graphs for the SMILE group.

**Figure 2:**
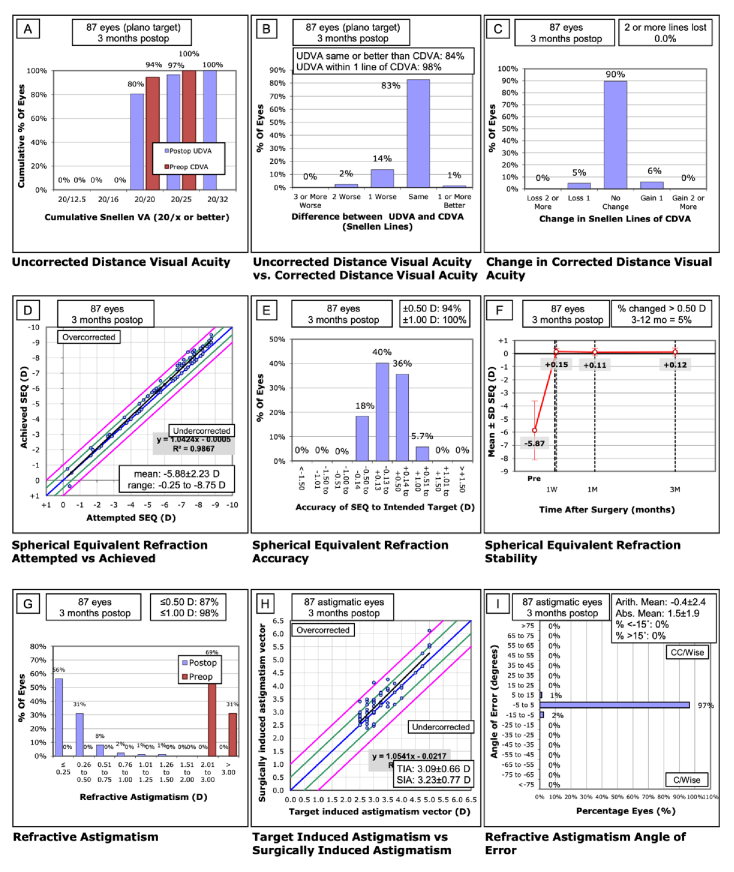
Standard graphs for the FS-LASIK group.

### Statistical Analysis

Statistical analysis was performed using SPSS version 26.0 (IBM Corp., Armonk, NY, USA). Intragroup comparisons were conducted using an independent *t*-test (parametric) or a Mann–Whitney U test (non-parametric). Statistical significance was set at *P* < 0.05. Standardized graphs were generated using the JRS report on refractive surgery.^[18]^ Vector analysis of astigmatism was performed using the Alpins method,^[20]^ with assistance from the AstigMATIC software.^[21]^ Vector parameters were calculated using the astigmatic vector analyzer application developed by Dr Alireza Peyman (https://www.drpeyman.ir/ophthweb/Ophthalmology_Calculator.htm).

The safety index was defined as the ratio of postoperative to preoperative CDVA, both expressed in decimal notation.^[22]^ It reflects the level of CDVA patients retain following surgery in comparison to the preoperative values.

##  RESULTS

A total of 170 eyes of 123 Vietnamese patients met the inclusion criteria and had 3-month postoperative data available for analysis. Eighty-three eyes (60 patients) underwent SMILE, and 87 eyes (63 patients) underwent FS-LASIK during the study period. The preoperative demographics of both groups are shown in Table 1. The number of females was higher in both groups: 75% of the participants in the SMILE group and 79% in the FS-LASIK group were female. There was no statistically significant difference between the two groups in age, cylinder, or BCVA (*P* > 0.05), but there was a statistically significant difference in sphere (Mann–Whitney U test, *P* < 0.05).

### Efficacy and Predictability

The nine standard graphs for the populations are shown in Figure 1 (SMILE) and Figure 2 (FS-LASIK). At the 3-month postoperative visit, there was no statistically significant difference between the two groups in terms of visual outcomes and residual manifest refraction. In the SMILE group, 90.4% of the eyes achieved a postoperative UDVA of 20/20 or better, whereas 98.8% of the eyes exhibited a preoperative CDVA of 20/20 or better. In the FS-LASIK group, 80.5% achieved a postoperative UDVA of 20/20 or better, while 94.3% had a preoperative CDVA of 20/20 or better. The percentage of eyes within ± 0.50 D of the intended manifest refraction spherical equivalent (SEQ) target was 95.1% after SMILE and 94.3% after FS-LASIK. The mean UDVA at the 3-month postoperative visit was 0.02 ± 0.05 logMAR for the SMILE group and 0.02 ± 0.04 logMAR for the FS-LASIK group. For astigmatism, 77% of eyes after SMILE and 86% of eyes after FS-LASIK were within ± 0.50 D of the intended target.

### Stability

The mean SEQ refraction was stable for up to 3 months after SMILE and FS-LASIK. After SMILE, the mean SEQ was +0.17 ± 0.37 D at 1 week, +0.11 ± 0.29 D at 1 month, and +0.13 ± 0.29 D at 3 months [Figure 1]. After FS-LASIK, the corresponding figures were +0.15 ± 0.27 D at 1 week, +0.11 ± 0.28 D at 1 month, and +0.12 ± 0.30 D at 3 months [Figure 2].

### Cylinder Vector Analysis

Table 2a and Table 2b show the comparative mean astigmatic values of the SMILE and FS-LASIK groups. There were no statistically significant differences in terms of TIA, DV, index of success (IOS), and angle of error (AE) between the two groups. However, statistically significant differences were found for SIA, correction index (CI), and magnitude of error (ME).

The SMILE group showed undercorrection of the cylinder, especially for the upper limit, at the 3-month postoperative visit [Figure 1]. The FS-LASIK group showed slight overcorrection of the cylinder, which was evenly balanced across the entire range of treatment [Figure 2]. The AE histogram showed that 96% of the eyes in the SMILE group [Figure 1] and 97% of the eyes in the FS-LASIK group [Figure 2] had an AE < 5º.

**Figure 3:**
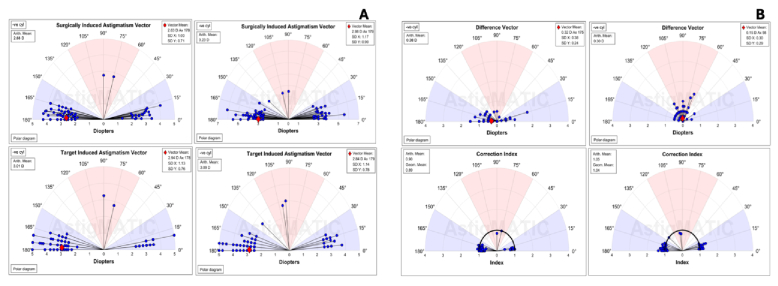
(a) The single-angle polar plot of TIA and SIA for both groups: Right: FS-LASIK group, Left: SMILE group. (b) The single-angle polar plot of DV and CI for both groups: Right: FS-LASIK group, Left: SMILE group.

Figure 3a shows the single-angle polar plots of TIA and SIA for both groups, while Figure 3b shows the single-angle polar plots of DV and CI for both groups. The polar plots with a vector means of TIA, SIA, DV, and CI at 3 months for the SMILE and FS-LASIK groups displayed both magnitude and axis values at each data point. Visually, both SMILE and FS-LASIK polar plots showed similar distributions of TIA, SIA, and CI. However, the two groups were different in terms of DV distributions, with the FS-LASIK group exhibiting astigmatic data points more concentrated in the against-the-rule area and of smaller magnitudes.

### Safety 

None of the eyes in the study experienced intraoperative complications, including suction loss or flap-related adverse events. We did not encounter any difficulties during femtosecond laser lenticule creation or manual dissection beneath the ink marks in any of the SMILE cases.

At the 3-month follow-up, there were no statistically significant differences in safety between the two groups. The safety index was 1.01 ± 0.05 for the SMILE group and 1.01 ± 0.09 for the FS-LASIK group (*P* = 0.374). In the SMILE group, 2.5% of the eyes gained one or more lines of CDVA, and there was no change in 96.30% of the eyes. There was a loss of one line of CDVA in one eye, and no eyes lost two or more lines of CDVA [Figure 1]. In the FS-LASIK group, 5.75% of eyes gained one or more lines of CDVA, and there was no change in 91.95% of the eyes. There was a loss of one line of CDVA in two eyes, and no eyes lost two or more lines of CDVA [Figure 2].

**Table 2a:** Visual and refractive outcomes at the 3-month postoperative visit

**Parameters**	**SMILE**	**FS-LASIK**	***P*** **-value**
UDVA (logMAR)	0.02 ± 0.05	0.02 ± 0.04	0.25
CDVA (logMAR)	0.004 ± 0.02	0.01 ± 0.03	0.04 ${}^{*}$
Safety	1.01 ± 0.05	1.01 ± 0.09	0.374
Efficacy	0.97 ± 0.10	0.96 ± 0.10	0.359
Sphere (D), mean ± SD	0.31 ± 0.28	0.26 ± 0.27	0.24
Cylinder (D), mean ± SD	0.39 ± 0.39	0.30 ± 0.33	0.12
SEQ	0.13 ± 0.29	0.12 ± 0.30	0.87
${}^{*}$ Significant difference, *P* < 0.05; SD, standard deviation; UDVA, uncorrected distance visual acuity; CDVA, corrected distance visual acuity; D, diopters; SEQ, spherical equivalent; logMAR, logarithm of the Minimum Angle of Resolution.			

**Table 2b:** Astigmatism parameters at the 3-month postoperative visit

**Parameters**	**SMILE (83)**	**FS-LASIK (87)**	**Ideal value**	***P*** **-value**
SIA, mean ± SD	2.68 ± 0.59 (1.56 to 4.08)	3.23 ± 0.77 (2.39 to 6.12)		< 0.01 ${}^{*}$
TIA, mean ± SD	3.01 ± 0.61 (2.50 to 4.75)	3.09 ± 0.66 (2.5 to 4.75)		0.559
DV, mean ± SD	0.38 ± 0.39	0.30 ± 0.33	0	0.155
CI, mean ± SD	0.89 ± 0.12	1.05 ± 0.1	1	< 0.01 ${}^{*}$
IOS, mean ± SD	0.13 ± 0.12	0.1 ± 0.11	0	0.091
ME, absolute, mean ± SD	0.34 ± 0.36	0.22 ± 0.27	0	0.02 ${}^{*}$
AE, absolute, mean ± SD	1.02 ± 1.81	1.50 ± 1.90	0	0.89
${}^{*}$ Significant difference, *P* < 0.05; SD, standard deviation; SIA, surgically induced astigmatism; TIA, target induced astigmatism; DV, difference vector; CI, correction index; IOS, index of success; ME, magnitude of error; AE = angle of error.				

**Table 3a:** Surgically induced and target-induced astigmatism values reported for patients with high myopic astigmatism

**Study**	**Group**	**SIA (D)**	**TIA (D)**
Zhou et al, 2022^[13]^	SMILE	2.18 ± 0.68	2.51 ± 0.56
	FS-LASIK	2.43 ± 0.73	2.65 ± 0.77
Zhao et al, 2021^[9]^	SMILE	2.37 ± 0.48	2.43 ± 0.52
	FS-LASIK	2.55 ± 0.60	2.57 ± 0.59
Chan et al, 2018^[14]^	SMILE	2.87 ± 0.47	2.94 ± 0.47
	FS-LASIK	2.97 ± 0.45	2.97 ± 0.44
Current study	SMILE	2.68 ± 0.59	3.01 ± 0.61
	FS-LASIK	3.23 ± 0.77	3.08 ± 0.66

**Table 3b:** Postoperative UDVA in SMILE and FS-LASIK reported for patients with high myopic astigmatism

**Study**	**Group**	**UCVA (logMAR)**
Zhou et al, 2022^[13]^	SMILE	- 0.05 ± 0.08
	FS-LASIK	- 0.03 ± 0.10
Zhao et al, 2021^[9]^	SMILE	0.06 ± 0.09
	FS-LASIK	0.06 ± 0.11
Chan et al, 2018^[14]^	SMILE	0.03 ± 0.05
	FS-LASIK	0.03 ± 0.04
Current study	SMILE	0.02 ± 0.05
	FS-LASIK	0.02 ± 0.04

**Table 3c:** Postoperative SEQ and astigmatism reported for patients with high myopic astigmatism

**Study**	**Group**	**SEQ (D)**	**Cyl (D)**
Zhou et al, 2022^[13]^	SMILE	- 0.10 ± 0.49	- 0.57 ± 0.40
	FS-LASIK	- 0.31 ± 0.63	- 0.46 ± 0.32
Zhao et al, 2021^[9]^	SMILE	-0.11 ± 0.19	- 0.19 ± 0.23
	FS-LASIK	- 0.10 ± 0.23	- 0.12 ± 0.26
Zhong et al, 2021^[24]^	SMILE (high astigmatism group)	- 0.23 ± 0.37	- 0.31 ± 0.29
	SMILE (low astigmatism group)	0.08 ± 0.49	- 0.20 ± 0.28
Chan et al, 2018^[14]^	SMILE	- 0.09 ± 0.22	- 0.27 ± 0.26
	FS-LASIK	- 0.02 ± 0.17	- 0.22 ± 0.22
Current study	SMILE	0.13 ± 0.29	- 0.39 ± 0.39
	FS-LASIK	0.12 ± 0.30	–0.30 ± 0.33

##  DISCUSSION

Both SMILE and FS-LASIK showed excellent safety and efficacy in the correction of high astigmatism. To the best of our knowledge, this is the first study comparing visual and refractive outcomes in eyes with high myopic astigmatism after SMILE and FS-LASIK in Vietnamese patients. The sample size is also one of the largest among the studies evaluating high astigmatism treatment. Chan et al^[16]^ and Zhou et al^[15]^ each performed retrospective analyses of patients who underwent SMILE and FS-LASIK for moderate-to-high astigmatism. Chan et al^[16]^ included 40 eyes that underwent SMILE and 65 eyes that underwent FS-LASIK with a follow-up duration of 3 months. Zhou et al^[15]^ included 53 eyes that underwent SMILE and 41 eyes that underwent FS-LASIK and followed them for 12 months. In the current study, the preoperative mean refractive cylinder was - 3.01 ± 0.61 D in the SMILE group and - 3.09 ± 0.66 D in the FS-LASIK group, which is higher than most other populations analyzed.^[23]^ Table 3a shows that TIA in both groups of the current study is higher than comparable groups in similar studies.

In the current study, there was no significant difference in visual outcomes or residual manifest refraction at the 3-month follow-up between the two groups. The mean UDVA was 0.02 logMAR in both groups, which compares favorably with results from similar studies [Table 3b]. The postoperative UDVA was 20/20 or better in 90.4% of the eyes in the SMILE group, compared to 98.8% of the eyes with a preoperative CDVA of 20/20 or better. The postoperative UDVA was 20/20 or better in 80.5% of the eyes in the FS-LASIK group, compared to 94.3% of the eyes with a preoperative CDVA of 20/20 or better. The satisfactory visual outcomes reflect minimal residual manifest refraction, which was comparable in both groups. Recently, Zhou et al^[15]^ found the postoperative SEQ to be closer to emmetropia after SMILE compared to FS-LASIK ( - 0.10 ± 0.49 vs. - 0.31 ± 0.63, respectively).

The postoperative cylinder values in the current study were also among the lowest compared with those in other studies [Table 3c]. According to Chan et al^[16]^ and Zhao et al^[9]^, the residual manifest cylinder was comparable between SMILE and FS-LASIK. While Chan et al^[16]^ reported a mean residual manifest cylinder of - 0.27 ± 0.26 for SMILE and - 0.22 ± 0.22 for FS-LASIK, Zhao et al^[9]^ noted a mean residual manifest cylinder of - 0.19 ± 0.23 for SMILE and - 0.12 ± 0.26 for FS-LASIK.

In the current study, SIA was significantly higher in the FS-LASIK group than in the SMILE group, while TIA was comparable between the two groups. This led to a higher CI for the FS-LASIK group and a higher absolute ME in the SMILE group. The absolute AE was lower in the SMILE group, although the difference did not reach statistical significance. The IOS was greater in the FS-LASIK group, but the difference was not statistically significant [Table 2b].

Eyes with a higher astigmatism magnitude have a greater likelihood of experiencing undercorrection following FS-LASIK,^[24]^ which aligns with the trend observed in previous studies reporting overcorrection in eyes with low astigmatism and undercorrection in eyes with high astigmatism.^[25,26]^ In one study, the authors reported an undercorrection of up to 21% of the attempted cylinder correction following the application of the MEL-80 excimer laser for FS-LASIK to treat high astigmatism (mean 3.9 D) in myopic eyes.^[25]^ Many previous studies^[9,15,16]^ reported that manifest astigmatism in the FS-LASIK group tends to be slightly undercorrected (with SIA being smaller than TIA and the CI being slightly below but close to 1). Studies comparing FS-LASIK with SMILE demonstrated similar residual astigmatism amplitudes between the two groups [Table 3c]. On the contrary, our results in the FS-LASIK group showed that manifest astigmatism was slightly overcorrected [Table 3a]. The polar plot of the DV vector showed that residual astigmatism in the SMILE group was predominantly with-the-rule, while it was mostly against-the-rule in the FS-LASIK group, confirming a higher overcorrection rate in the FS-LASIK group.

In the SMILE group, SIA was lower than TIA (2.68 ± 0.59 D vs. 3.01 ± 0.61 D, respectively), resulting in a small degree of undercorrection. This was reflected in a mean CI of 0.89 ± 0.12 [Table 2b], which is comparable to the results of the high astigmatism group reported by Zhong et al,^[27]^ who found an average CI of 0.94. In contrast, the FS-LASIK group exhibited an SIA greater than TIA (3.23 ± 0.77 D vs. 3.09 ± 0.66 D, respectively), leading to slight overcorrection and a CI of 1.05 ± 0.10 [Table 2b]. Since all eyes in the study had preoperative with-the-rule astigmatism, the overcorrection in the FS-LASIK group resulted in residual against-the-rule astigmatism, a known consequence of excessive flattening along the vertical meridian.

In this study, DV, IOS, and AE did not differ significantly between SMILE and FS-LASIK when the procedures were performed by the same surgeon [Table 2b], suggesting that both techniques achieved comparable precision in astigmatic correction under consistent surgical conditions. However, ME was significantly higher in the SMILE group compared to the FS-LASIK group (0.34 D vs. 0.22 D, *P* = 0.02), indicating a greater tendency toward undercorrection in SMILE. Nevertheless, AE was comparably low in both groups (*P* = 0.89), with correction indices of 0.89 for SMILE and 1.05 for FS-LASIK.

Theoretically, vector analysis shows that axis misalignment leads to a reduced flattening effect: a 5º misalignment results in a 1.5% loss, 15º in 13.4%, and 30º in up to 50%.^[28]^ Accurate correction of astigmatism depends heavily on proper alignment of the treatment axis, particularly in high myopic astigmatism, where even a 5º deviation can meaningfully affect outcomes.^[29]^ In the current study, excellent axis alignment was maintained during both SMILE and FS-LASIK procedures, resulting in consistently low AE values. The mean AE in the SMILE group was 1.01º—the lowest reported in comparative studies to date—and notably lower than the values reported by Zhang et al (absolute AE = 2.24º, mean TIA = 2.48 D),^[30]^ and Ganesh et al (AE = 2.31º, mean TIA = 2.19 D).^[31]^ This observation further supports the precision of SMILE in maintaining optimal treatment orientation.

The DV in the SMILE and FS-LASIK groups (0.38 D and 0.30 D, respectively, *P* = 0.16) showed no significant difference between the two procedures in the current study. This finding is consistent with previous reports: Chan et al reported DV values of 0.27 D for SMILE and 0.22 D for FS-LASIK (*P* = 0.34),^[16]^ while Zhou et al reported 0.59 D for SMILE and 0.46 D for FS-LASIK (*P* = 0.07), and comparable outcomes for CI and IOS, with slightly higher DV and lower CI in the SMILE group compared to the FS-LASIK group.^[15]^ Other studies have also demonstrated similar levels of residual astigmatism between the two techniques [Table 3c]. Taken together, these results suggest that undercorrection occurs more frequently in SMILE, despite comparable vector accuracy with the other technique.

One of the possible causes of the decreased accuracy of astigmatic correction with SMILE is the lack of automatic intraoperative compensation for cyclotorsion with VisuMax 500. The surgeon needed to mark the patient's cornea in an upright position and then manually adjust the treatment cone during docking. However, there have been several reports that show excellent cylinder correction with SMILE when using preoperative limbal marking.^[31,32,33,34]^ Another way to improve the accuracy of astigmatic correction is to develop a personalized nomogram, especially for high cylinders. Recently, Yu et al developed a nomogram based on a regression between TIA and flattening effect, and they found enhanced accuracy and predictability for high myopic astigmatism correction ( > 2.25 D) in SMILE surgery.^[35]^ Moreover, a 10% overcorrection in the magnitude of astigmatism has been recommended to offset undercorrection tendencies observed in patients.^[36,37,38,39]^

The current study showed comparable safety and efficacy indices. Chan et al^[16]^ showed that there was no statistically significant difference between SMILE and FS-LASIK at the 3-month follow-up, with mean safety indices of 0.99 (SMILE) and 0.98 (FS-LASIK) and efficacy indices of 0.95 (SMILE) and 0.94 (FS-LASIK). Zhou et al^[12]^ showed a similar safety index (SMILE = 1.14 vs. FS-LASIK = 1.19) and efficacy index (SMILE = 1.17 vs. FS-LASIK = 1.16).

The predictability of SEQ within ± 0.50 D of the intended target at 3 months was 95% and 94% in the SMILE and FS-LASIK groups, respectively, which is similar to that reported by Chan et al^[16]^ at 3 months (SMILE = 97% and FS-LASIK = 100%) but higher than that reported by Zhou et al^[15]^ at 12 months (SMILE = 85% and FS-LASIK = 66%). Excellent stability was found in the SMILE and FS-LASIK groups up to 3 months in the current study, which is consistent with the study by Chan et al.^[16]^

The limitations of the study include its retrospective design and the limited follow-up period (3 months), which is relatively short for assessing refractive stability. In addition, as part of the standard clinic protocol, neither preoperative nor postoperative visual acuity was pushed beyond 20/20. It is crucial to consider this factor when comparing our findings with those of other studies. In the future, we will conduct a prospective randomized comparative study incorporating standardized visual acuity measurements and a longer follow-up period to validate our results and evaluate potential differences in regression between groups. An updated nomogram that incorporates a greater number of patients with a high cylinder will also be employed for both treatment groups based on the analysis of this initial study.

To the best of our knowledge, no previous study has compared SMILE and FS-LASIK for correction of high astigmatism in a Vietnamese population using vector analysis. This study suggests that SMILE and FS-LASIK have comparable efficacy and safety in correcting high myopic astigmatism.

##  Financial Support and Sponsorship

None.

##  Conflicts of Interest

None.
